# Postnatally-transmitted HIV-1 Envelope variants have similar neutralization-sensitivity and function to that of nontransmitted breast milk variants

**DOI:** 10.1186/1742-4690-10-3

**Published:** 2013-01-10

**Authors:** Genevieve G Fouda, Tatenda Mahlokozera, Jesus F Salazar-Gonzalez, Maria G Salazar, Gerald Learn, Surender B Kumar, S Moses Dennison, Elizabeth Russell, Katherine Rizzolo, Frederick Jaeger, Fangping Cai, Nathan A Vandergrift, Feng Gao, Beatrice Hahn, George M Shaw, Christina Ochsenbauer, Ronald Swanstrom, Steve Meshnick, Victor Mwapasa, Linda Kalilani, Susan Fiscus, David Montefiori, Barton Haynes, Jesse Kwiek, S Munir Alam, Sallie R Permar

**Affiliations:** 1 Human Vaccine Institute, Duke University Medical Center, Durham, NC, USA; 2 Department of Medicine, University of Alabama at Birmingham, Birmingham, AL, USA; 3 Department of Medicine, University of Pennsylvania, Philadelphia, PA, USA; 4 Department of Microbial Infection and Immunity, Ohio State University, Columbus, Ohio, USA; 5 Department of Microbiology and Immunology, University of North Carolina, Chapel Hill, NC, USA; 6 Department of Epidemiology, University of North Carolina, Chapel Hill, NC, USA; 7 Division of Viral Pathogenesis, Beth Israel Deaconess Medical Center, Boston, MA, USA; 8 Department of Community Health, University of Malawi, Blantyre, Malawi

**Keywords:** HIV, Mother to child transmission, Galcer, Dendritic cells, Neutralizing antibodies

## Abstract

**Background:**

Breastfeeding is a leading cause of infant HIV-1 infection in the developing world, yet only a minority of infants exposed to HIV-1 via breastfeeding become infected. As a genetic bottleneck severely restricts the number of postnatally-transmitted variants, genetic or phenotypic properties of the virus Envelope (Env) could be important for the establishment of infant infection. We examined the efficiency of virologic functions required for initiation of infection in the gastrointestinal tract and the neutralization sensitivity of HIV-1 Env variants isolated from milk of three postnatally-transmitting mothers (n=13 viruses), five clinically-matched nontransmitting mothers (n=16 viruses), and seven postnatally-infected infants (n = 7 postnatally-transmitted/founder (T/F) viruses).

**Results:**

There was no difference in the efficiency of epithelial cell interactions between Env virus variants from the breast milk of transmitting and nontransmitting mothers. Moreover, there was similar efficiency of DC-mediated trans-infection, CCR5-usage, target cell fusion, and infectivity between HIV-1 Env-pseudoviruses from nontransmitting mothers and postnatal T/F viruses. Milk Env-pseudoviruses were generally sensitive to neutralization by autologous maternal plasma and resistant to breast milk neutralization. Infant T/F Env-pseudoviruses were equally sensitive to neutralization by broadly-neutralizing monoclonal and polyclonal antibodies as compared to nontransmitted breast milk Env variants.

**Conclusion:**

Postnatally-T/F Env variants do not appear to possess a superior ability to interact with and cross a mucosal barrier or an exceptional resistance to neutralization that define their capability to initiate infection across the infant gastrointestinal tract in the setting of preexisting maternal antibodies.

## Background

Mother to child transmission (MTCT) of HIV-1 via breastfeeding is responsible for over a third of pediatric HIV-1 infections in the developing world [1]. These postnatal infections occur throughout the duration of breastfeeding [2,3]. Interestingly, in the absence of antiretroviral prophylaxis, less than 10% of breastfed infants born to HIV-1-infected women acquire HIV-1 [2], despite many months of exposure to large quantities of milk containing cell-free and cell-associated virus [4,5]. As high levels of maternal plasma virus load and low CD4 cell counts only partially account for the risk of infant transmission [6], the virion-host interactions required for this inefficient postnatal transmission remain largely undefined. As with other routes of MTCT [7-10], there is a genetic bottleneck that restricts the number of virus variants transmitted through breastfeeding to a single or a small number of variants [11]. A comparative analysis of the genotypic and phenotypic characteristics of postnatally-transmitted and nontransmitted HIV-1 variants is critical for understanding the biologic mechanisms of postnatal HIV-1 transmission and designing targeted prophylactic strategies.

Several groups have reported genetic differences between transmitted and chronic HIV-1 Env variants [12-15]. For example, heterosexually-transmitted clade C Env variants have fewer putative N-linked glycosylation sites, more compact variable loops, and are more sensitive to autologous neutralization [13]. Similarly, Env variants from infants infected during delivery usually have shorter variable loops and fewer glycosylation sites than maternal variants [14]. Interestingly, the analysis of a large number of clade B T/F *env* gene sequences has recently led to the identification of putative transmission signature sequences in the CCR5 binding site and gp160 signal peptide [16], however, the functional significance of these transmitted virus signature sequences remains ill-defined [17]. Mucosal transmission of clade B HIV-1 viruses has also been associated with CD4+ T cell tropism and efficient CCR5 usage [18-20]. A superior ability of virions to perform key steps required for mucosal invasion, such as high efficiency binding to mucosal epithelial cells or enhanced ability to be transferred by sub-epithelial DCs to CD4+ T cells in the sub-mucosa or lymphoid tissue could confer a selective advantage to HIV-1 variants during postnatal transmission.

Novel anti-HIV-1 monoclonal antibodies (mAbs) capable of neutralizing a broad spectrum of HIV-1 isolates have recently been isolated [21-24] and could be useful tools for passive immunization or for the design of active immunization strategies to prevent MTCT. A protective role of broadly-neutralizing antibodies in breast milk HIV-1 acquisition has been established in non-human primates studies, as passive infusion of broadly-neutralizing mAbs protected neonatal rhesus monkeys against oral challenge with a simian-human immunodeficiency virus [25,26]. However, previous studies have indicated that viruses transmitted during breastfeeding are typically resistant to neutralization by maternal autologous plasma and broadly-neutralizing antibodies [11,27-29]. Nevertheless, the neutralization breadth of maternally- acquired HIV-specific antibodies does not appear to correlate with infant protection from postnatal HIV-1 acquisition [30]. Furthermore, Env variants from breast milk and plasma appear to be equally-sensitive to autologous neutralization [31]. Thus, a better understanding of the neutralizing phenotype of breast milk viruses of postnatal-transmitting women, including their sensitivity to the new generation of broadly neutralizing mAbs, will help design immunologic interventions to prevent postnatal HIV-1 acquisition.

While previous studies investigated the neutralization phenotype of postnatally-transmitted viruses [11,32], no previous studies have compared the genotype and phenotype of breast milk Env variants from transmitting and nontransmitting mothers. Moreover, previous investigations of infant T/F Env variants phenotype have not included the assessment of the ability to interact with and cross a mucosal barrier. Efficient interaction with epithelial cells or tissue-associated DCs may be required for HIV-1 transmission in the gastrointestinal tract. In this study, we compare the genotype and function of 30 clade C Env variants isolated from the breast milk of eight HIV-infected women who did or did not transmit HIV-1 to their infants during breastfeeding and of 6 T/F Env variants isolated from postnatally-infected infants. Defining a phenotype of postnatally-transmitted virus variants will guide the development of immunologic interventions to reduce HIV-1 transmission via breastfeeding.

## Results

### Selection of env variants from breast milk of postnatally-transmitting and nontransmitting mothers and from plasma of postnatally-infected infants

From a cohort of HIV-1-infected lactating women (CHAVI 009) [33], HIV-1 *env* gene sequences were amplified by SGA from milk collected at 4 to 6 weeks after delivery from mothers who were confirmed to postnatally-transmit HIV-1 to their infant (n = 3). Postnatal infection was defined by a negative infant whole blood HIV-1 DNA PCR at birth and four weeks of age and a positive dried blood spot and/or whole blood HIV-1 DNA PCR at three and/or six months of age. HIV-1 *env* gene sequences were also amplified from the milk of five nontransmitting HIV-1-infected, lactating mothers (defined by a negative infant whole blood HIV-1 DNA PCR at 9 months of age, following weaning, and all prior time points) from the same cohort, matched for maternal CD4 count and HIV-1 milk RNA viral load (Table 1).The plasma virus load 4–6 weeks after delivery ranged from 3,070 to 100,892 RNA copies/ml in transmitting women and from 35,200 to 359,000 RNA copies/ml in nontransmitting women (p=0.14). Similarly, there was no statistical difference in the milk virus load of transmitting and nontransmitting women (milk virus RNA copies/ml range 635 to 101,500 versus 1,315 to 31,900 in left breast and 615 to 30,450 versus 265 to 22,500 in right breast in transmitting and nontransmitting women respectively, p=0.77 and 0.39). The range of the plasma and milk virus loads was similar to that observed in other studies [34]. The antibody response in the plasma and milk of the study participants has been previously reported [31].

**Table 1:** Maternal and infant clinical parameters

	**Subject**	**Blood CD4 count/ml**	**Plasma VL RNA copies/ml**	**Milk VL RNA copies/ml (L and R)**	**Age of infant at HIV+ PCR**
**Postnatally-transmitting mothers**	**0404**	80	22,600	29,650	6 mo
				29,200	
	**1209**	153	3,070	635	6 mo
				615	
	**4403**	208	100,892	101, 500	3 mo
				30,450	
**Nontransmitting mothers**	**0301**	406	159,000	1,315	n/a
				265	
	**0702**	523	35,200	31,900	n/a
				2,530	
	**3305**	166	359,000	3,335	n/a
				16,150	
	**4707**	247	83,400	10,500	n/a
				22,500	
	**5807**	188	138,000	2,760	n/a
				1,970	

HIV-1 *env* gene sequences were analyzed by maximum-likelihood tree phylogenetic analysis [33]. Breast milk HIV-1 *env* sequences without frameshift mutations or in-frame stop codons representing distinct branches on the phylogenetic trees were selected for cloning (Additional file 1: Figure S1). When possible, *env* sequences were selected from a group of identical or nearly identical *env* sequences, as these sequences likely represent clonally-amplified, functional viruses that recently-infected a local target cell [33].

**Figure 1:**
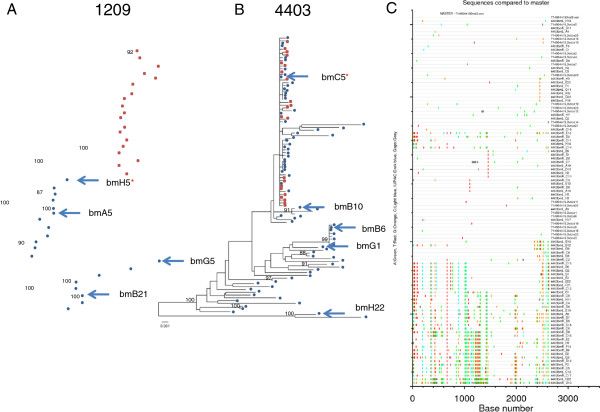
**Phylogenetic comparison of virus *env *sequences from the plasma of postnatally-infected infants (red squares) and paired mother’s milk (blue circles).** The infant of maternal subject 1209 (**A**) was first HIV DNA positive at 6 months of age, but a plasma sample was not available for virus sequencing until 12 months of age, thus infant virus evolution away from the transmitted maternal variant had occurred. The infant of maternal subject 4403 (**B**) was first HIV DNA positive at 3 months of age and infant plasma virus sequencing was performed from a sample collected at the same timepoint. The consensus of the infant plasma *env* sequences was confirmed to be identical to the maternal breast milk variant 4403 bmC5 by highlight plot (**C**). The postnatally-T/F variant from subject 4403 and the maternal milk *env* variant most closely related to the transmitted variant from subject 1209 are indicated with a red star.

Two of the three postnatally-infected infants born to the postnatally-transmitting mothers had adequate plasma available following confirmation of infection in the CHAVI 009 study for SGA amplification and sequencing of the infant plasma HIV-1 *env* sequences (Figure 1). The infant of subject 4403 had plasma available at the same time point as the first positive HIV-1 DNA PCR (3 months of age) (Figure 1B), whereas the infant of subject 1209 did not have plasma available for sequencing until 12 months of age, 6 months after the initial positive HIV-1 DNA PCR (Figure 1A). These infant sequences were phylogenetically compared to the breast milk sequences from the transmitting mother. The milk *env* variant of subject 4403 that was identical to the T/F infant variant was well-defined to be 4403 bmC5 via phylogenetic (Figure 1B) and highlighter plot (Figure 1C) analysis; this virus is part of a large group of identical and nearly identical viruses in the milk of this mother [33]. The maternal milk *env* variant in transmitting mother 1209 that was most closely related to the *env* sequences present in the infant six months following infection was 1209 bmH5 (with a genetic distance of 1.4% from the matched infant consensus virus sequence). Thus, 4403 bmC5 milk variant represents the infant postnatally T/F virus of 4403 and is included in the analysis of infant postnatally-T/F viruses, whereas 1209 bmH5 milk variant only represents the sequenced milk variant most closely related to the virus transmitted to the 1209 infant.

Finally, the remaining six infant postnatally-T/F Env variants were amplified from plasma or PBMCs collected at 12 weeks of age of six Malawian infants who were plasma HIV-1 DNA negative at birth and six weeks of age, yet positive at 12 weeks of age, as described elsewhere [35]. The cloned consensus of the infant *env* sequences represented an inferred consensus sequence of a virus present at or near the time of HIV-1 transmission, based on the Poisson-Fitter analysis, and thus are referred to as infant T/F viruses [19].

### Previously-defined T/F virus *env* signature sequence patterns in env gene sequences from postnatal-transmitting and nontransmitting mothers and postnatally-infected infants

The presence of previously-defined putative T/F virus *env* gene sequence signature patterns was assessed in the *env* gene sequences of viruses cloned from the breast milk of postnatally-transmitting and nontransmitting mothers and from blood-derived *env* gene sequences of six unpaired postnatally-infected infants, including: the absence of a threonine at position 415 (H415) and the absence of a histidine at position 12 in the Env signal sequence [16]. Neither of these genetic signatures, defined in clade B adult T/F viruses, was common in the maternal or infant virus variants in our cohort. Genetic signatures identified in the sieve analysis of *env* V2 sequences from the RV144 HIV vaccine trial [36] were also assessed. Most postnatal T/F and milk variants had a lysine residue at position 169 (7 of 7 postnatal T/F and 32 of 36 milk Env variants) and an isoleucine residue at position 181 (6 of 7 postnatal T/F and 29 of 36 milk Env variants) in the V2 loop, matching that of the RV144 vaccine insert. We then investigated the presence of an isoleucine at position 6 of the Env sequence that have been reported to be prevalent in clade C *env* genes isolated from HIV infected women who transmitted HIV *in utero* as compared to nontransmitting women [37]. An isoleucine at position 6 was observed in 12 of 13 (92%) viruses from milk of postnatal-transmitting mothers, and in 5 of 7 (71%) postnatally-T/F infant viruses but in only 12 of 23 (52%) viruses of nontransmitting mothers. There was no difference in variable loop length (median in transmitting mothers: 144, median in nontransmitting mothers: 146, p=0.9) or number of potential N-linked glycosylation sites (median in transmitting mothers: 28.5, median in nontransmitting mothers: 31, p=0.7) between milk *env* variants from women who transmitted or did not transmit HIV-1 to their infants during breastfeeding.

### Breast milk Env variants from postnatal-transmitting and nontransmitting mothers display similar efficiency of binding to colonic epithelial cells and the putative HIV-1 epithelial cell attachment factor, galatosyl ceramide (Galcer)

**Figure 2:**
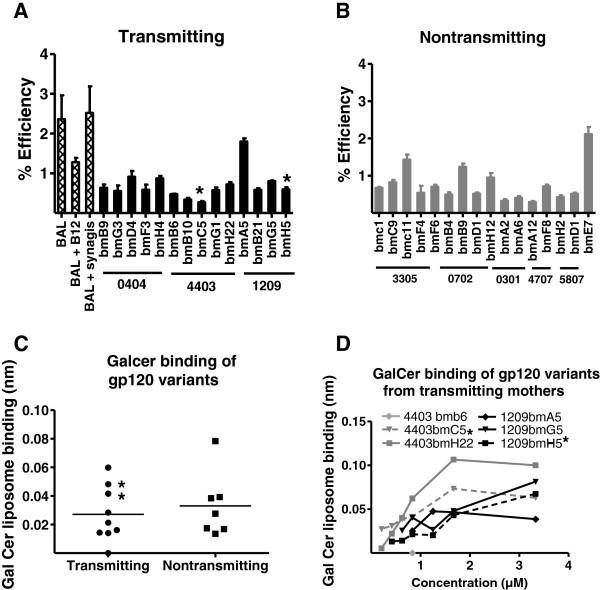
**No difference in binding to colonic epithelial cells or galactosyl ceramide (Galcer) between Env variants from women who did and did not postnatally transmit HIV-1 to their infants.** Binding of milk Env variants from postnatal-transmitting (**A**) and nontransmitting (**B**) mothers to colonic epithelial HT-29 cells. (**C**) Binding of gp120 proteins expressed by milk Env virus variants to Galcer measured by biolayer interferometry. (**D**) Binding of gp120 variants from transmitting mothers to Galcer. Stars represent transmitted variants, error bars represent standard deviation and lines represent means of two experiments performed in triplicate.

The initial steps of establishing a productive infection at a mucosal surface requires HIV-1 virions to attach to and cross a mucosal epithelial cell barrier. Thus, we sought to determine if HIV-1 Env pseudovirus variants from milk of women who postnatally-transmitted the virus to their infant were more efficient in their ability to attach to columnar gastrointestinal epithelial cells (HT-29 cells) than those from women who did not transmit the virus postnatally. We first determined if the binding of Env variants to HT-29 cells was Env dependent by assessing binding inhibition of HIV BAL by the anti-HIV mAb IgG1B12 (25 ug/ml). The anti-RSV mAb synagis was used a negative control. B12 inhibited HIV BAL interaction to epithelial cells with a range of 40 to 60% as compared to synagis, indicating that the interaction is a least partially Env dependent (Figure 2A). The efficiency of breast milk HIV-1 Env pseudovirus variant attachment to columnar epithelial cells was between 0.3 and 2.1% and did not differ between postnatally-transmitting (Figure 2A) and nontransmitting women (Figure 2B) (p = 0.9). Moreover, neither the postnatally-T/F Env variant (4403 bmC5) nor the breast milk Env variant most closely related to the postnatally-transmitted variant (1209 bmH5) identified in the milk of transmitting women had an unusually high efficiency to attach to columnar epithelial cells (Figure 2A).

Next, we assessed the ability of the gp120 Env proteins expressed by the milk Env variants that had the highest efficiency at binding to columnar epithelial cells (n = 9 Env variants from 3 transmitting mothers and 7 Env variants from 3 nontransmitting mothers) to bind to Galcer, the putative HIV epithelial cell attachment factor [38]. Again, there was no difference in the efficiency of the gp120 proteins expressed by HIV variants in milk of transmitting and nontransmitting mothers to bind to Galcer (Figure 2C). Moreover, the transmitted Env variants did not have the strongest binding to Galcer when compared to nontransmitted variants from the same women (Figure 2D). Thus, we did not find evidence that the ability of a virus Env to associate with colonic epithelial cells or Galcer was a defining feature of viruses in milk of postnatal-transmitting mothers.

### Postnatally-transmitted Env variants are efficiently transferred from mature DCs to CD4-expressing target cells

**Figure 3:**
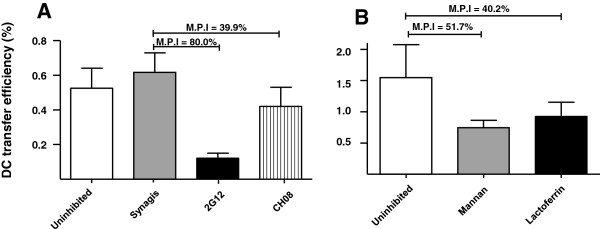
**DC transfer of breast milk HIV Env variants is at least partially Env mediated and is inhibited by lectin-binding molecules, including human lactoferrin.** (**A**) Virions were incubated with a blocking agent (anti-Env monoclonal antibody, mannan, or lactoferrin) then incubated with mature DCs prior to inoculation of TZM-bl cells. DC transfer inhibition of a plasma Env virus variant from a transmitting mother by anti-HIV Env mAbs 2 G12 and CH08 (A) relative to a non-HIV-specific control antibody, Synagis (α-RSV) (**B**). Inhibition of DC transfer of a postnatally-transmitted infant-derived Env virus variant by α-methyl-D-mannopyranoside (C_7_H_14_0_6_) and by human lactoferrin, relative to the respective uninhibited virus controls. Bars represent averages of three replicates and error bars represent standard deviations. Mean percent inhibition (M.P.I) values relative to the appropriate controls are indicated.

The next early step that is likely to be important in establishing productive mucosal infection is the interaction of HIV with DCs in the sub-epithelium of mucosal surfaces [39]. Virus-bound DCs are thought to traffic virions to lymphoid tissue where they can productively infect CD4+ T cells. DC trans-infection of CD4+ T cells appears to be dependent on interaction with the HIV Env, as DC-mediated transfer to CD4-expressing reporter cells was inhibited by the glycan-dependent anti-HIV-1 Env mAb 2 G12, as well as a CD4-inducible HIV-neutralizing mAb isolated from colostrum B cells CH08 [40] (Mean Percent Inhibition (M.P.I) of 80.0 and 39.9%, respectively) relative to an irrelevant anti-RSV mAb, Synagis (Figure 3A). Breast milk is a rich source of anti-lectin glycans and glycoproteins such as lactoferrin and high mannose glycans [41,42], that have been shown to interrupt the interaction of HIV Env with DC lectin binding molecules [43,44]. Therefore, we assessed the ability of lactoferrin (milk concentration 2.9 mg/ml, range 1.3 to 6.9 mg/ml) [45] and α-methyl-D-mannopyranoside (C_7_H_14_O_6;_ free mannose milk concentration 42 ± 33 μM) [46] to inhibit the DC-transfer of an infant T/F virus variant. Both reagents were inhibitory when compared to the uninhibited virus control with an M.P.I of 40.2% and 51.7% for lactoferrin and C_7_H_14_O_6_, respectively (Figure 3B), confirming that breast milk containing carbohydrates are able to inhibit DC-HIV interactions.

**Figure 4:**
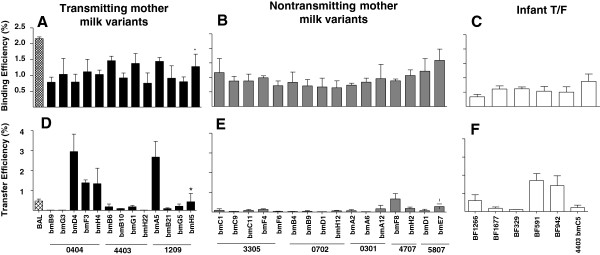
**DC binding and transfer of breast milk and postnatally-acquired T/F infant Env variants.** DC binding (**A**-**C**) and transfer (**D**-**F**) efficiencies of breast milk Env virus variants from transmitting (**A**, **D**) and nontransmitting (**B**, **E**) mothers and infant-derived Env virus variants (**C**,**F**). Bars represent the averages of three replicates from two independent experiments. The star represents the milk Env variant most closely related to the postnatally-transmitted breast milk Env variant in subject 1209.

As the efficient virion interaction with DCs in the sub-epithelial in the setting of carbohydrate-rich milk could be a defining feature of postnatally-transmitted Env variants, we sought to determine if postnatally-transmitted HIV Env pseudovirus variants were more efficient in their ability to bind to DCs and subsequently be transferred to permissive CD4+ cells compared to nontransmitted variants. The efficiency of breast milk and infant HIV-1 Env pseudovirus binding to DCs ranged between 0.3 and 1.6% (Figure 4A-C) and the efficiency of DC-mediated trans-infection ranged between 0.03 and 2.9% (Figure 4D-F). While there was no difference in the average binding efficiencies of breast milk Env pseudovirus variants from postnatally-transmitting and nontransmitting women (p = 0.45), the postnatally-T/F Env pseudoviruses were less efficient at binding to DCs compared to the average DC-binding efficiency of milk variants from nontransmitting women (p = 0.008). However, this difference only trended towards significant when correcting for multiple comparisons (q value 0.06). This difference in binding efficiency of infant Env variants and nontransmitted milk variants did not appear to be attributable to interaction with DC-SIGN, as Env variant binding to DC-SIGN-expressing cells both in the presence and absence of mannan was similar between infant T/F variants and breast milk Env variants (Additional file 2: Figure S2).

Finally, we sought to determine if the relatively low DC-binding efficiency of infant T/F variants affected their ability to be transferred to CD4+ cells. Despite the low DC binding-efficiency of the postnatally-T/F infant Env pseudovirus variants, these infant viruses had DC transfer efficiencies that trended towards higher than that of Env variants from milk of nontransmitting women (p = 0.06) (Figure 4F). However, breast milk variants of transmitting and nontransmitting mothers had similar DC-transfer efficiency (p = 0.10). We next assessed the relationship between the number of glycosylation sites on the Env protein and the efficiency of Env variant DC-binding and transfer. There was no correlation between the DC binding efficiencies of the breast milk and infant Env pseudovirus variants and the number of Env potential N-linked and O-linked glycosylation sites (r=0.1, p = 0.6 and r=−0.07, p = 0.7 respectively), nor was there a correlation between the transfer efficiencies and the number of N-linked glycosylation sites (r=−0.2, p = 0.3). In contrast, the transfer efficiencies of the breast milk and infant Env pseudovirus variants positively correlated with the number of Env O-linked glycosylation sites (r=0.4 p = 0.03), indicating a potential link between the Env amino acid sequence, glycosylation pattern, and DC-transfer efficiency.

As the glycosylation pattern of Env virions can be influenced by the cell producing type [47], we compared the DC transfer potency of infectious molecular clones (IMCs) expressing the breast milk virus *env* variants 4403 bmC5 and 1209 bmH5 and produced either in peripheral blood mononuclear cells (PBMC) or in 293 T cells. The mean transfer efficiency of the 4403 bmC5 IMCs produced in PBMC and 293 T cells was 0.3% (range 0.1 to 0.6) and 0.5% (range 0.1 to 0.6), respectively. The mean transfer efficiency was also very similar for the 1209 bmH5 IMCs (PBMC: mean 0.6%; range 0.3 to 1.4 and 293 T: mean 0.5%, range 0.1 to 0.9). This data suggests that the *in vitro* DC-mediated virus transfer efficiency of the breast milk HIV Env variants produced in 293 T is representative of viruses produced in host primary cells.

### No difference in efficiency of viral entry between Env variants from milk of postnatally-transmitting and nontransmitting mothers and plasma of postnatally-infected infants

**Figure 5:**
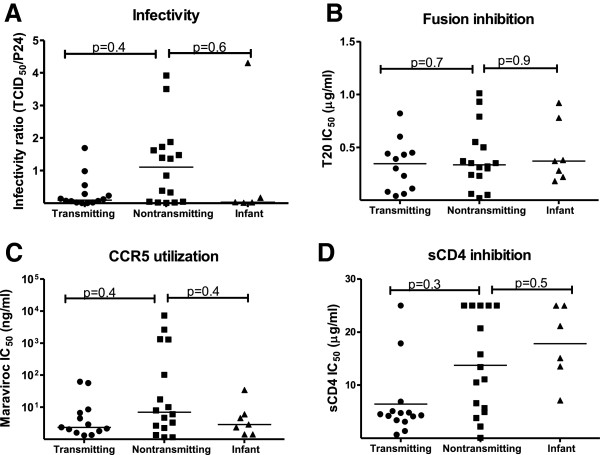
**No difference in the efficiency of viral entry between Env virus variants from the milk of transmitting and nontransmitting women, or postnatally infected infants.** (**A**) Infectivity ratio (TCID_50_/ml divided by p24 amount in ng/ml) of Env virus variants; (**B**) Inhibition of Env virus variant infectivity by CCR5 antagonist Maraviroc; (**C**) Inhibition of membrane fusion by entry inhibitor T20 peptide. Lines represent means IC50s (performed in duplicate for each virus) for each group of Env variants.

We next investigated potential differences in viral entry and efficiency of coreceptor usage between milk Env variants from postnatally-transmitting and nontransmitting women and postnatally-infected infants. First, we determined the infectivity of the Env pseudovirions in TZM-bl cells. To control for the efficiency of the transfection reaction employed to produce the Env pseudoviruses, the infectivity ratio was calculated by dividing the TCID_50_/ml determined in TZM-bl reporter cells by the p24 content of each Env pseudovirus stock [33] (Figure 5A). There was no difference in the infectivity of Env pseudoviruses from the milk of transmitting mothers when compared to those in milk of nontransmitting women (p=0.38). Similarly the infectivity of Env variants from postnatally infected infants was comparable to that of Env variants from the milk of nontransmitting women (p=0.64). There was also no difference in the ability of the fusion inhibition T20 peptide to inhibit the infectivity of Env pseudovirions from either transmitting mothers (p=0.75) or infant (p=0.9) as compared to Env variants from nontransmitting mothers (Figure 5B). As previous studies have reported a bias toward CCR5 usage in T/F viruses [19,48], we investigated the coreceptor tropism of breast milk variants and infant T/F variants. All the Env variants had a CCR5 tropism as determined by a reduction/ absence of TZM-bl cell infection in the presence of the CCR5 antagonist TAK-779, but not in the presence of the CXCR4 antagonist AMD-3100 (data not shown). The efficiency of CCR5 utilization by the Env variants was assessed by incubating TZM-bl cells increasing concentrations of the CCR5 antagonist, Maraviroc prior to infection (Figure 5C). The mean IC_50_ of Maraviroc was not different between Env variants from infants or transmitting mothers and variants from nontransmitting mothers (p=0.36 and p=0.36). Finally, to study the efficiency of CD4 engagement and postnatal HIV transmission, we measured inhibition of the milk and infant Env pseudoviruses infectivity by soluble CD4 (sCD4) and CD4-Ig. There was no statistical difference in the mean sCD4 IC_50_ of Env variants of transmitting and nontransmitting women (p= 0.27) (Figure 5D). Similarly, infant Env viruses variants were comparably sensitive to sCD4 as nontransmitting Env variants (p= 0.45). Thus, postnatally-transmitted Env variants do not appear to be more efficient at viral entry than viruses in milk of nontransmitting mothers.

### Env variants from milk of postnatally-transmitting and nontransmitting mothers and postnatally-infected infants are equally-sensitive to neutralization by autologous plasma and milk

Early studies of vertically-transmitted viruses indicated that viruses transmitted during breastfeeding are generally resistant to neutralization by autologous maternal plasma [11,27]. Therefore, we measured autologous plasma and milk neutralization of Env pseudoviruses from milk of transmitting mothers and nontransmitting mothers. Because breast milk from uninfected women possesses low potency HIV-1 inhibitory activity [49,50], breast milk neutralization was considered positive if the neutralization titer was greater than 3 standard deviation above the mean ID_50_ of three HIV-negative milk samples. Eleven of 14 milk Env pseudoviruses from transmitting mothers were neutralized by autologous plasma (Table 2) and 13 of 15 milk Env variants from nontransmitting mothers were neutralized by autologous plasma (p=0.65), whereas only two milk Env variants from transmitting women and four milk Env variants from nontransmitting women were neutralized by autologous milk (p=0.65). Interestingly, the most potent autologous neutralization activity was observed against the milk Env variant that is related to the transmitted variant from the milk of subject 1209 (1209 bmH5) (Figure 1), with ID_50_ of 2079 and 202 in plasma and milk, respectively. In contrast, the T/F variant in milk of a transmitting mother (4403 bmC5) was relatively resistant to neutralization by autologous plasma (ID_50_=25) and milk (ID_50_<5). Together, our data suggest that viruses in the milk of postnatal-transmitting women are not inherently more resistant to neutralization by autologous maternal plasma or milk than viruses from the milk of nontransmitting women.

**Table 2:** Neutralization of breast milk viruses by autologous plasma and breast milk

		**Plasma**	**Breast Milk**^1, 2^	**Uninfected milk1**	**Uninfected milk 2**	**Uninfected milk 3**
**3305 BMC1**	Nontransmitting	***20***	<5	6	16	12
**3305 BMC9**	Nontransmitting	***38***	<5	10	17	26
**3305 BMC11**	Nontransmitting	***22***	<5	5	15	11
**3305 BMF4**	Nontransmitting	<20	5	12	48	32
**3305 BMF6**	Nontransmitting	***30***	6	12	15	39
**0301 BMA2**	Nontransmitting	***45***	5	5	5	26
**0301 BMA6**	Nontransmitting	***24***	4	<5	8	31
**0301 BMA12**	Nontransmitting	***83***	31	24	8	86
**4707 BMF8**	Nontransmitting	***42***	***56***	8	19	21
**4707 BM H2**	Nontransmitting	***32***	27	14	23	17
**702 BMB4**	Nontransmitting	<20	***77***	18	9	20
**702 BMB9**	Nontransmitting	***26***	***54***	17	17	28
**702 BMH12**	Nontransmitting	***36***	***56***	22	29	32
**5807 BMD1**	Nontransmitting	***44***	<5	7	<5	<5
**5807 BME7**	Nontransmitting	***24***	<5	7	<5	<5
**4403 BMB6**	Transmitting	***28***	<5	<5	12	6
**4403 BMB10**	Transmitting	***30***	<5	<5	12	6
**4403 BMC5**	Transmitting	***25***	<5	<5	12	10
**4403 BMG1**	Transmitting	***22***	<5	<5	15	5
**4403 BMH22**	Transmitting	***117***	***53***	<5	10	<5
**1209 BMA5**	Transmitting	***451***	39	44	9	8
**1209 BMB21**	Transmitting	***50***	13	41	10	9
**1209 BMG5**	Transmitting	***429***	22	50	10	19
**1209 BMH5**	Transmitting	***2079***	***202***	22	7	<5
**0404 BMB9**	Transmitting	***27***	9	24	7	6
**0404 BMD4**	Transmitting	***33***	16	42	17	16
**0404 BMF3**	Transmitting	<20	7	64	16	10
**0404 BMG3**	Transmitting	<20	<5	10	6	35
**0404 BMH4**	Transmitting	<20	<5	15	<5	<5

^1^Neutralization above the cut off of 3 standard deviations above the inhibitory dilution 50% of milk of uninfected mothers or plasma from uninfected individuals is bold and italic. ^2^Numbers indicate inhibitory dilution 50%.

### Breast milk Env variants from transmitting women and T/F postnatal infant Env variants are equally-sensitive to neutralization by broadly-neutralizing monoclonal and polyclonal antibodies as milk Env variants of nontransmitting mothers

**Figure 6:**
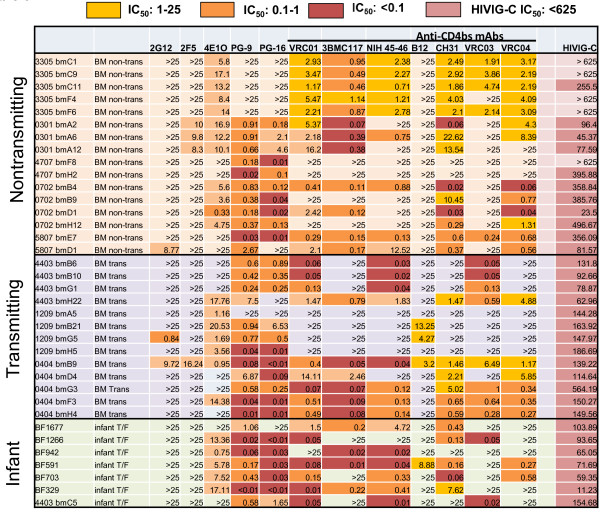
Neutralization sensitivity of Env variants from breast milk of postnatally transmitting or nontransmitting women and of infant T/F Env variants to broadly neutralizing polyclonal and monoclonal antibodies.

We next examined the sensitivity of milk and infant Env pseudoviruses to broadly-neutralizing mAbs including 4E10, 2F5, 2G12, PG9 and PG16 (Figure 6). As previously reported for clade C viruses [51,52], most of the Env variants were resistant to neutralization by 2F5 and 2G12 at the highest tested concentration of 25 μg/ml (Figure 6). Only three viruses (two from a transmitting and one from a nontransmitting mother) were neutralized by 2G12 and 4 viruses were neutralized by 2F5 (one from a transmitting and three from nontransmitting mothers). In contrast, most Env variants were sensitive to neutralization by the other mAbs (Figure 6). There was no statistical difference in the median IC_50_ of 4E10 between Env variants from either infants or milk of transmitting mothers as compared to variants from milk of nontransmitting mothers (p= 0.88 and p=0.39 respectively). Similarly, the median IC_50_ of PG9 and PG16 was not different between either Env variants from postnatally infected infants (p= 0.56 and p=0.24 respectively) or transmitting women (p=0.67 and p=0.4, respectively) and those of nontransmitting women. The sensitivity of the mother and infant Env variants to HIVIG-C, a pool of HIV-1 specific IgG from clade C infected individuals with broad neutralizing activity was also evaluated. All Env variants from transmitting mothers were neutralized by HIVIG-C whereas Env variants from two of five nontransmitting women had milk viruses that were resistant to neutralization at the highest concentration of HIVIG-C tested (625 μg/ml). Moreover, infant T/F Env variants were significantly more sensitive to neutralization by HIVIG-C than milk variants from nontransmitting mothers (median IC_50_ 71.69 μg/ml versus 255.5 μg/ml, p= 0.01). However, this difference only trended towards significance when correcting for multiple comparisons (q = 0.06). Thus, the postnatally-transmitted infant viruses in our cohort were relatively sensitive to neutralization by broadly-neutralizing polyclonal and monoclonal Abs.

### Postnatally-transmitted infant viruses are sensitive to neutralization by broadly-neutralizing mAbs against the CD4 binding site

It was recently reported that T/F clade B viruses are more sensitive to neutralization by anti-CD4 binding site (bs) mAbs b12 and VRC01 than chronic viruses [20]. Therefore, we investigated the sensitivity of postnatal T/F infant and breast milk Env variants to inhibition by a panel of broadly-neutralizing mAbs against the CD4 bs including b12, CH31, 3BNC 117, NIH 45–46, VRC01, VRC03 and VRC04 (Figure 6). Binding of the mAbs 3BNC117, NIH45-46 and VRC01 to the CD4 bs induces a conformational change that enhances the binding of CD4-induced mAbs (such as 17B) to their epitopes in the co-receptor-binding region of gp120; whereas CH31, VRC03, VRC04 and b12 do not induce this conformational change [21,53]. xEnv pseudoviruses from milk of transmitting and nontransmitting mothers were equally sensitive to neutralization by VRC01 and CH31 (p = 0.81 and 0.83, respectively). Moreover, postnatal-T/F variants were also equally sensitive VRC01 and CH31 compared to milk Env variants from nontransmitting women (p = 0.45 and 0.9, respectively). Importantly, 6 of 7 T/F infant viruses were neutralized by VRC01 and NIH 45–46 (Figure 6), suggesting that postnatally-transmitted infant viruses are generally sensitive to neutralization by anti-CD4 bs broadly-neutralizing mAbs. The sensitivity of the majority of infant viruses to the new generation of anti-CD4 bs mAbs indicates that these reagents could be useful in the prevention of postnatal HIV-1 transmission.

## Discussion

Assessing the genetic and functional characteristics of transmitted HIV-1 Env variants isolated from donors and recipients is important to identify any biological signatures associated with mucosal transmission that can be exploited in the design of prophylaxis strategies. To gain insights into the biologic properties of viruses capable of breaching the infant gastrointestinal mucosa during transmission through breastfeeding, we isolated and produced 30 Env variants from the breast milk of 8 HIV-1-infected women who did or did not transmit HIV-1 to their infants postnatally and investigated their genetic and phenotypic characteristics. In addition, T/F Envs from 7 infants infected through breastfeeding were also studied. This represents one of the largest phenotypic comparative analyses of postnatally-T/F variants and uniquely compares their virologic phenotype to nontransmitted breast milk Env variants.

Several studies have attempted to identify genetic differences between chronic and transmitted viruses, with disparate results. The majority have reported that acute viruses have shorter variable loops and fewer glycosylation sites than chronic viruses [9,13,14,54]. However, while comparing sequences from clade B chronic (n=24) and T/F *envs* (n=17), Wilen and collaborators [20] found no genetic signatures associated with transmission. Interestingly, a larger sequence analysis of clade B *env* gene sequences allowed for the identification of a small number of signature sequences that could influence Env incorporation and viral entry [16]. In this study, we found that based on the Env V2 sequence, all infant T/F variants and most maternal milk variants are likely to have been susceptible to the potentially-protective V2-specific antibody responses elicited by the RV144 vaccine. Moreover, we observed that a previously-identified unique sequence (isoleucine at position 6) in the signal sequence of the *env* gene of placentally-transmitted clade C variants [37] was common in Env variants of postnatal-transmitting women and postnatally-infected infants, although the observed high frequency of this mutation could be due to the relatively small number of mothers and infants included in the study. The location of this signature amino acid in the signal sequence of the Env gene is not likely to affect the structure or interactions of the Env protein, but could affect the efficiency of Env production. Association of an Env signature sequence with more than one MTCT route may indicate that this unique sequence provides a fitness advantage to the virus in the infant [12]. Therefore, it would be important to analyze this putative genetic signature in the *env* signal sequence in a larger panel of both clade B and C MTCT T/F *envs*.

The ability of HIV-1 virions to perform key steps required to cross the infant gastrointestinal epithelial barrier could contribute to restricting the number of founder viruses. We tested this hypothesis by measuring the interaction of milk Env variants with intestinal epithelial cells. Further, we assessed the ability of gp120 from these Envs to bind to Galcer, the putative epithelial cell attachment factor for HIV-1 [38]. Milk Env variants from transmitting and nontransmitting women had an equally low efficiency epithelial cell attachment and Galcer binding. These results are in accordance with previous reports of low level of transcytosis across epithelial barriers [55-57]. However, previous studies have reported that Galcer binds efficiently to both gp120 and gp41 [58-60]. The interaction of the HIV Env with Galcer may be dependent on conformational epitopes and multivalent interactions. Thus, future work should investigate the binding of monomeric and polymeric gp140 of transmitted variants to Galcer liposomes.

Previous studies have indicated that DCs are present in infant oral and intestinal sub-mucosa [61]. The binding of HIV virions to these cells and their transfer to CD4+ cells may be an important step in the establishment of mucosal HIV-1 infection [39], especially in the setting of postnatal transmission, as carbohydrates in milk may compete for interaction with lectin-binding molecules on DCs [43,62]. While there appeared to be lower DC-binding efficiency of postnatally-T/F Env variants compared to those in milk of nontransmitting women, the efficiency of DC-mediated virus transfer was not different between the transmitted and nontransmitted variants. The direct correlation between DC-mediated trans-infection efficiency and number of O-linked glycosylation sites suggests that this function may more dependent on glycan:DC interactions. In this study TZM-bl cells were used as target cells instead of primary CD4 + T cells, which may better represent *in vivo* function, in order to assure reproducibility and comparability across assays. Our results are similar to those of Wilen and collaborators [20] who reported no difference DC trans-infection efficiency between chronic and T/F clade B viruses from sexual transmission. Therefore, the ability of transmitted Env variants from other MTCT transmission routes and other virus subtypes should be investigated to determine if efficient DC to CD4+ T cell transfer is a particular feature of HIV-1 MTCT. Of note, the Env pseudoviruses and recombinant Env proteins used in these studies were primarily produced in 293 T cells, which may yield distinct glycosylation patterns compared to primary cells. Thus, these investigations mainly address functional differences in transmitted and nontransmitted Env variants at the amino acid level. However, a comparison of DC transfer efficiency of two breast milk full length, replication-competent Env variants revealed similar transfer efficiencies, suggesting that the Env variants produced in 293 T cells are representative of those produced in primary host cells in this assay.

Infant and adult T/F viruses poorly infect monocyte-derived macrophages (MDM) [18,63] and primarily utilize CCR5 [18-20,48]. In accordance with previous reports, all the postnatal T/F Env variants were CCR5-tropic viruses, and there was no difference in CCR5 utilization between transmitted and nontransmitted milk variants. Moreover, CD4 utilization and infectivity in TZM-bl cells were comparable between transmitted and nontransmitted milk variants. Thus, our results suggest no difference in viral entry mechanisms between infant postnatal T/F and milk nontransmitted Env variants.

Maternal antibodies are transferred to the fetus during pregnancy and, at birth, infants born to HIV-1 infected women have high levels of HIV-specific antibodies [30,64]. Furthermore, HIV Env-specific functional antibodies are present in the milk of HIV-infected women [31,65]. These maternal antibodies may provide some protection from HIV acquisition [32,65-67]. However, studies have found conflicting results regarding associations between maternal and infant antibody responses and MTCT, probably related to differences in modes of transmission and virus clades being assessed [30,68]. Maternal neutralizing antibodies may also exert selective pressure on viruses that could contribute to the transmission bottleneck. In support of this hypothesis, previous studies reported that viruses transmitted during breastfeeding are usually resistant to maternal autologous neutralization [27,32]. However, other studies did not observe increased resistance of infant viruses to neutralization by maternal plasma [14,63,69,70]. Furthermore, no difference in sensitivity to autologous neutralization was observed between clade C viruses from postnatally-infected infant and viruses from their mothers [28]. Accordingly, we also found no difference in autologous milk or plasma neutralization of milk Env variants from women who did or did not transmitted HIV to their infants postnatally. In addition, most Env variants from women who transmitted HIV to their infants through breastfeeding and from postnatally infected infants were sensitive to neutralization by the new generation of highly-potent broadly neutralizing mAbs (PG9, PG16, VRC01, and CH31). Thus, our results, together with other recent reports, suggest that neutralization-resistance may not be a defining feature of HIV-1 clade C viruses transmitted during breastfeeding.

Our inability to discern major differences in the genotype and phenotype of postnatal-T/F variants could support that crossing the epithelial barrier is a stochastic event that does not depend on specific biologic properties of the Env. Fortunately, animal studies have established that infusion of broadly-neutralizing antibodies can protect the infant against postnatal HIV-1 acquisition [26]. Thus, an infant vaccine to prevent HIV-1 transmission through breastfeeding would have to induce rapidly-elicited protective antibodies to ensure safe breastfeeding. Interestingly HIV vaccination of infants born to HIV infected women induces immune responses distinct from that of their mothers [71]. As infant immune response could take weeks to be established, maternal vaccination to elicit antibody responses in breast milk and/or passive infusion of the infant with broadly-neutralizing, protective mAbs could assure undisrupted protection of infants throughout the breastfeeding period. Interestingly, passive immunotherapy with broadly-neutralizing mAbs against the CD4 binding site in infant rhesus monkeys was shown to accelerate the development of functional antibody responses following SIV infection [72]. Thus, passive administration of specific mAbs prior to infant vaccination might also be beneficial for the induction of the rapid immune responses needed in the setting of breast milk transmission.

## Conclusions

We have investigated the biologic characteristics of 36 HIV-1 variants from either the breast milk of HIV-1 infected mothers or from postnatally-infected infants. We did not find a genotypic or phenotypic characteristic unique to infant postnatal T/F viruses. Similarly, no clear genetic or functional differences were observed between milk Env variants of women who transmitted or did not transmit HIV postnatally to their infants. Importantly, most infant Env variants (6–7 out of 7) were neutralized by VRC01, PG9, and PG16, indicating that they are good candidates for infant passive immunization. Thus, these mAbs should be investigated for their ability to prevent postnatal HIV-1 transmission.

## Methods

### Study population

Pregnant women testing HIV-positive by rapid antibody test were recruited from two rural health clinics outside Blantyre, Malawi as part of the CHAVI 009 protocol between November 2007 and November 2008, as previously described [31,33]. Women were enrolled at delivery if HIV-1 infection was confirmed by detection of plasma HIV-1 RNA and breastfeeding was initiated. Except for single dose nevirapine at delivery, the women included in this study remained off antiretroviral therapy during lactation. The mothers were counseled to exclusively breastfeed and weaned their infants between 6 and 9 months of age, per WHO recommendations during that time. HIV-1 *env* gene variants were sequenced and cloned from milk and infant plasma collected four to six weeks post-delivery from three women who transmitted HIV-1 postnatally to their infants and from five HIV-infected women, matched by peripheral CD4 count and breast milk HIV-1 RNA load, who did not transmit the virus to their infant. *Env* gene sequences from six unpaired Malawian infants from the Malaria and HIV-1 in Pregnancy prospective cohort [9,73-76] infected with HIV-1 during breastfeeding (HIV-1 DNA negative by real-time PCR at 0 and 6 weeks, positive at 12 weeks) were also included [35]. Both studies were approved by the College of Medicine Research and Ethics Committee in Malawi and institutional review boards at each of the participating institutions where samples were received or processed for end-user analysis.

### HIV-1 *env* gene single genome amplification and cloning

Breast milk supernatant aliquots of 1 ml were concentrated 10 fold by centrifugation at 23,600 × g for 1 h at 4°C (final volume 100 μl), and RNA was extracted. Depending on the milk virus RNA load, between 200 and 10,000 RNA copies were reverse transcribed. Plasma viral RNA and cell-associated DNA were also extracted from blood samples collected 12 weeks after delivery from postnatally-infected infants. Single-strand cDNA was synthesized using the SuperScript III protocol according to the manufacturer’s instructions (Invitrogen Life Technologies) and either used immediately for PCR or stored frozen at −80°C. Full-length *env* genes were PCR amplified by single genome amplification (SGA) methods as previously described [19]. Briefly, cDNA was titrated by endpoint dilution (undiluted to 1:40 dilution for breast milk cDNA) in 96-well PCR plates to a concentration that yielded no more than 30% PCR positive wells and conforms to a Poisson distribution of a single template per reaction. All milk *env* sequences used in this study are deposited in GenBank [accession numbers HM070449 to HM070824 and HQ595810 to HQ596189].

### Phylogenetic sequence analysis

Breast milk *env* sequences showing significant evidence of APOBEC-driven G-to-A hypermutation, according to the Los Alamos National Laboratory (LANL) HIV Sequence Database tool Hypermut 2.0 [77] (http://www.hiv.lanl.gov/content/sequence/HYPERMUT/hypermut.html), were excluded from the phylogenetic analyses. Sequence alignments were produced using ClustalW [78] and were subsequently adjusted to optimize codon alignment. Regions that could not be aligned unambiguously were omitted in further phylogenetic analyses but were included in all other genetic and phenotypic analyses of the virus populations. A phylogenetic tree including sequences from all 8 subjects indicated that the sequences corresponding to each subject clustered with high bootstrap support (100% [data not shown]) [78]. For each subject, a maximum likelihood phylogenetic tree was inferred using PhyML, version 3.0 [79], and the results of approximate likelihood ratio tests (≥0.95) were used to infer phylogenetic support [80]. For the infant plasma *env* sequences, the number of viruses and genetic identity of the *env* gene responsible for establishing productive clinical infection in each infant was determined by SGA and a mathematical model of random evolution, as previously described [33] using the Poisson-Fitter tool (http://www.hiv.lanl.gov/content/sequence/POISSON_FITTER/poisson_fitter.html). Briefly, infant plasma *env* sequences (n=9-29 amplicons per sample) were highly homogeneous in all infants by highlighter plot analysis (http://www.hiv.lanl.gov/content/sequence/HIGHLIGHT/highlighter_top.html), except one infant (infant of 1209), in which plasma was not available for SGA until 6 months after infection, and conformed to a model of random evolution and coalesced to a single consensus. The consensus of the T/F *env* sequence in the postnatally-infected infant plasma at twelve weeks of age was cloned into pcDNA3.1 directional TOPO expression vector (Life Technologies), grown in TOP10 competent cells, sequence confirmed, and used in transfection of 293 T cells to generate Env variant pseudovirions. The number of days that would be required by the most recent common ancestor to reach the *env* diversity observed in the 12-week infant blood sample was also calculated using the Poisson-fitter tool. Based on model predictions, the selected infant *env* gene was calculated to have been transmitted <90 days from the time the plasma sample was obtained, conforming to the timing of these postnatal transmissions [35].

### Sequence genotype/phenotype analysis

Assessment of previously-defined T/F Env signature patterns [16] was accomplished by alignment of the infant and maternal *env* sequences with the HXB2 reference sequence using the HIValign program at the LANL website (http://www.hiv.lanl.gov/content/sequence/VIRALIGN/viralign.html) for standard nucleotide position numbering. Potential N-linked sites were enumerated using the N-glycoSite tool from the Los Alamos National Laboratory (LANL) website (http://www.hiv.lanl.gov/content/sequence/GLYCOSITE/glycosite.html) which locates putative N-glycosylation sites in an amino acid sequence. An estimate of the number of O-linked glycosylation sites was carried out using the NetPhos program from the Center for Biological Sequence Analysis website (http://www.cbs.dtu.dk/services/NetPhos/). Putative phosphorylation sites with a predicted score greater than 0.5 were defined as probable O-linked glycosylation sites.

### Env pseudoviruses preparation

Env pseudoviruses were prepared by transfection in 293 T cells with 4 μg of *env* plasmid DNA and 8 μg of *env*-deficient HIV-1 plasmid DNA (SG3Δenv) using the FuGENE 6 transfection reagent (Roche Diagnostic). For autologous neutralization assays, a mutated backbone resistant to reverse transcriptase inhibitors (1617RT/K103N, obtained from Ron Swanstrom) was used. Two days after transfection, the culture supernatant containing pseudoviruses was harvested, filtered, aliquoted and stored at −80°C. An aliquot of frozen pseudovirus was used to measure the infectivity in TZM-bl cells. Serial five-fold dilutions of pseudovirus were distributed in quadruplicates to 96 well flat bottom plates (Costar) in a total volume of 100 μl per well. Then, freshly trypsinized TZM-bl cells were added (10,000 cells/ well in DMEM 10%FBS containing HEPES and 10 μg/ml of DEAE-dextran). After 48 hours of incubation at 37°C, 100 μl of cells/well were transferred to 96-wells black solid plate (Costar) and the luminescence measured using the Bright Glo luminescence reporter gene assay system (Promega). Wells producing relative luminescence units > 3x background TZM-bl luminescence were scored as positive and the TCID_50_ was calculated by the method of Reed and Muench, as previously described [81]. Out of a total of 43 Env variants produced, only HIV-1 Env pseudoviruses that produced functional infectious Env-pseudotyped variants (TCID_50_ > 1000/ml in TZM-bl reporter cells) (n = 14 from transmitting mothers, n = 16 from nontransmitting mothers and n=6 from postnatally infected infants) were used in functional assays.

### Epithelial cell binding

A modified protocol from Mantis *et al*. [82] was used to measure the binding of Env variants to the colonic cell line HT-29 (ATCC). Briefly, HT-29 were grown to confluence on collagen-treated 96 well flat bottom plates in Modified McCoy’s 5a Medium supplemented with 10% Fetal Bovine Serum (FBS) and antibiotics. The cells were washed twice, then Env variants diluted in serum-free media were added in triplicate and the plates were incubated at 37°C for 3 hours. To determine the amount of virus bound to cells, the cells were washed 3 times with DPBS (Gibco) to remove free virus, detached with TrypLE (Invitrogen), and lysed with Triton-X 100. Levels of p24 were measured in cell lysates by commercial ELISA (Perkins-Elmer) according to the manufacturer protocol. Binding efficiency was calculated by dividing the amount of p24 recovered by the p24 input measured in the same ELISA. Results from two independent assays performed in triplicate were averaged. For Env dependency assay, HIV BAL was incubated either with growth media, mAb IgG1B12 (25 μg/ml) or the control antibody synagis (25 μg/ml) for 1 hour at 37°C prior to addition to the cells.

### Production of gp120 Env variants

Codon-optimized gp120 *env* genes were commercially-synthesized (Genewiz, Inc.) and cloned into the pcDNA3.1+ expression vector (Invitrogen). The expression vectors containing *env* gene inserts were used for polyethyleneimine-mediated transfection of 293 T cells. The resulting recombinant Env gp120 glycoproteins were purified from supernatants of transfected 293 T cell cultures using *Galanthus nivalis* lectin-agarose column chromatography (Vector Labs) and analyzed by SDS-polyacrylamide gel electrophoresis and by western blot with the anti-HIV-1 Env monoclonal antibody VRC C 16H3. Monomers of the gp120 proteins were isolated from their oligomers by Fast Protein Liquid Chromatography (FPLC) and stored at −70°C until use.

### Binding of gp120 Env variants to Galcer

The lipids 1- palmitoyl-2-oleoyl-sn-glycero-3-phosphocholine (POPC) and D-galactosyl-β-1,1’ N-octanoyl-D-erythro-sphingosine (Galcer) (Avanti Polar Lipids) were used to make liposomes using extrusion technique, as described earlier [83,84]. Appropriate volumes of stock solutions of POPC (dissolved in chloroform) or Galcer (dissolved in chloroform:methanol 70:30 v/v) and POPC in a 1:1 molar ratio were mixed . The lipid mixture was dried in a stream of gaseous nitrogen and any residual solvent was removed by drying the lipids film under a vacuum overnight. The dried lipid mixture was hydrated with PBS buffer (pH 7.4) and incubated at 60°C for 45 minutes, prior to sonication with an ultrasonic liquid processor (Misonix, Inc), and extrusion through 0.4 μm and 0.1 μm polycarbonate membranes using a mini-extruder (Avanti Polar Lipids). The binding of breast milk HIV-1 gp120 monomers to Galcer was performed by Biolayer Interferometry Technique using a ForteBio OctetRed instrument and Aminopropylsilane (APS) biosensors. Briefly, the Galcer and POPC liposomes (250 μM placed in the sample plate well) were loaded onto APS biosensors by dipping them into sample plate wells containing 250 μM of liposomes for 10 minutes. As a control, an APS sensor was dipped into a well containing PBS buffer. The blank and liposome-loaded APS sensors were washed in PBS for 1 minute. In order to minimize non-specific interaction of breast milk HIV-1 gp120 monomers with the blank and liposome-loaded APS sensors, the sensors were coated with bovine serum albumin (BSA) by dipping into wells containing 0.1% BSA for 5 minutes followed by a wash with PBS for 1 minute. The interaction of HIV-1 gp120 monomers with Galcer liposomes was measured by monitoring wavelength shift (in nanometer). After 2 minutes dip in PBS to obtain baseline, the blank and liposomes-loaded APS sensors were dipped into wells containing gp120 monomers at a 100 μg/ml (or at the indicated) concentration for 30 minutes followed by a 10 minutes wash in PBS. The signal from blank APS sensor was subtracted to obtain signal specific to Galcer and POPC liposome binding. The gp120 monomers either did not bind POPC liposomes or bound extremely weakly. Any such weak binding to POPC liposomes was subtracted from the Galcer liposome binding to obtain Galcer specific binding.

### Generation of virus stocks in 293 T cells and PBMCs

*Renilla* Luciferase expressing replication competent HIV-1 proviral DNA was constructed as previously described [85] to express the breast milk virus *env* variant sequences, 4403 bmC5 and 1209 bmH5. The plasmids were used to transfect 293 T cells using the FuGENE 6 transfection reagent (Roche Diagnostic). The cell culture supernatant containing viruses was harvested after 48 and 72 hours, filtered, aliquoted and stored at −80°C. Viruses produced in 293 T were used to infect human PBMC that have been activated overnight with PHA-P. After 24 hours, the culture supernatant was removed and fresh medium was added. Virus production was monitored by p24 production and the cell culture supernatant was harvested was the p24 amount was ≥ 10 ng/ml, filtered, aliquoted and stored at −80°C. Virus stocks produced in 293 T cells and PBMC were evaluated for infectivity in TZM-bl cells.

### DC binding and transfer

Peripheral Blood Mononuclear cells (PBMCs) were isolated by ficoll-paque gradient centrifugation of fresh whole blood from three to five donors per assay. A total of 50 × 10^6^ PBMCs per plate were cultured in 10-cm tissue culture plates for 1-hr to initiate adherence selection of monocytes. Cells from different donors were cultured separately to avoid mixed leukocyte reactions. Culture dishes were subsequently washed to remove non-adherent cells. The monocytes were induced to differentiate into immature dendritic cells (DC) by culturing them in the presence of 200 IU/ml of IL-4 and 100 IU/ml GM-CSF for 5 days. The cytokines were refreshed on day 2 or 3 of incubation. Immature DCs were harvested on Day 5 and plated in 6-well plates at a concentration of 1.5 × 10^6^ cells per well. Lipopolysaccharide (LPS) at a concentration of 50 ng/ml was added as a maturation stimulus on day 6 and mature DCs were harvested on day 7.

On Day 6 of the DC maturation process, TZM-bl reporter cells were plated in 96 well plates. The reporter cells were obtained through the NIH AIDS Research and Reference Reagent Program, Division of AIDS, NIAID, NIH (Dr. John C. Kappes, Dr. Xiaoyun Wu and Tranzyme Inc). On Day 7, DCs were harvested, washed, and resuspended in fresh media at 2,500,000 cells/ml for binding assays and at 3,000,000 cells/ml for transfer assays. A 100 μl aliquot of the cell suspension was added to each experimental well. Triplicate wells were set up for each experimental condition. 20–25 ng of p24 of each Env pseudotyped virus, in a total volume of 100 μl, was then added to each well. The DCs and virus were co-cultured at 37°C for 2 hours. For antibody inhibition assays, the virus was pre-incubated with 30 μg/mL monoclonal antibody (mAb) (anti-HIV-1 Env mAb 2 G12 [86], CH08 [40] or anti-Respiratory Syncytial Virus (RSV) mAb Syangis) for 30 minutes prior to co-culturing with the DCs. This concentration is similar to the concentration of total IgG in breast milk in this population (median 80 μg/ml range: 20 to 400 μg/ml [31]. For the Env competition assay, the JRFL Env gp120 was pre-incubated with the DCs for 30 minutes at 37°C prior to addition of the Env pseudovirus. For the anti-lectin treatments with methyl-α-D-mannopyranoside (C_7_H_14_O_6_; Sigma-Aldrich) and human lactoferrin (Sigma-Aldrich), the DCs were pre-incubated with 0.5 M C_7_H_14_O_6_ or 100 μg/mL lactoferrin prior to addition of the Env pseudovirus. After the 2 hour incubation, the cells were washed 3 times with media to remove unbound virus. To measure DC-binding efficiency, the cells were lysed with 0.5% Triton-X 100 prior to quantification of the amount of bound virus using a commercial p24 ELISA kit according to the manufacturer’s recommendations (Perkins-Elmer, Inc.). Binding efficiencies were calculated by dividing the amount of DC-bound p24 by the original p24 amount of the stock virus added to the experimental well, measured in the same p24 ELISA. To assess DC transfer efficiency, 11 two-fold serial dilutions of the washed DCs were prepared in final volumes of 100 μl. The initial DC dilution had a total of 150,000 cells/100 μl. The eleven DC dilutions for each experimental condition were added to a 96-well plate with plated TZM-bl cells, with the last well in each row serving as a virus-free experimental background control. Following 48 hour of co-culture incubation at 37°C, DC trans-infection of the TZM-bl cells was detected using Bright-Glo luciferase assay system (Promega). The 50% Tissue culture infectious dose (TCID_50_) for each experimental condition was calculated using the method of Reed and Muench, with a cutoff for positive wells set at two times the average of the background wells for each respective plate. Transfer efficiencies were calculated by dividing the TCID_50_ of the pseudovirus-loaded DCs by the TCID_50_ of the stock pseudovirus used in the experiment. Infectious pseudoviruses with undetectable DC transfer efficiencies (n = 5) were assigned a transfer efficiency halfway between zero and the lowest transfer efficiency detected in the study (0.027%) for statistical analysis.

### DC-SIGN mediated trans-infection

Parental 3 T3 and a 3 T3 cell line stably expressing human DC-SIGN were obtained from the NIAID AIDS Research and Reference Reagent Program [87]. Approximately 10,000 3 T3-DC-SIGN cells or parental 3 T3 cells were plated for 24 hours in 100uL DMEM containing 10% FBS (Sigma). Prior to addition of HIV-1 Env pseudoviruses, cells were pretreated with 2 μg/μL Mannan solution (Sigma) or DMEM media (Invitrogen) alone for 30 minutes at 37°C. Next, 5 ng p24 Env-pseudovirus was added and cells were spinoculated for 2 hrs at 1000 X g at 37°C. Following spinoculation, the cells were washed with 200μL of media and co-cultured with 10,000 TZM-bl cells for 48 hours. Co-cultured cells were then washed with 100μL PBS, lysed with 1X Reporter Lysis Buffer (Promega), and frozen at −80°C. Cells were frozen and thawed three times prior to enumeration of luciferase activity. Luciferase activity was monitored in GloMax 96 Microplate Luminometer (Promega) per manufacturer’s instructions. All experiments were performed in triplicate and data was normalized to the Env-pseudovirus TCID_50_. The mannan-sensitive DC-SIGN-mediated transfer was calculated by subtracting the transfer efficiency in the presence of mannan from that in the absence of mannan.

### Coreceptor usage

Coreceptor usage was determined in TZM-bl cells by using TAK-779 (NIH HIV reagent repository), a CCR5 receptor antagonist, and AMD-3100 (NIH HIV reagent repository), a CXCR4 antagonist. Freshly trypsinized TZM-bl cells were distributed into 96-well plates (10,000 cells/well in DMEM-10% FBS containing HEPES and 10 μg/ml of DEAE-dextran) and incubated for 1 hour with either TAK-779 (10 μM), AMD-3100 (1.3 μM), a combination of the two reagents, or no inhibitor (control wells). Pseudoviruses were added to wells with the different treatments in triplicate. After 48 hours of incubation, luminescence was measured. Wells containing coreceptor inhibitors were compared to control wells to determine if either agent led to a reduction in infectivity. Viruses known to use either CCR5 or CXCR4 were used a controls. For testing the efficiency of CCR5 utilization, diluted preparations of the CCR5 antagonist Maraviroc [88] were incubated with TZM-BL cells for 1 hour then Env variants were added. After 48 hours, luciferase expression was measured (Bright-Glo, Promega) and the IC_50_ was calculated.

### Fusion inhibition

The efficiency of viral entry by the different Env variants was assessed in TZM-bl cells. Briefly, diluted preparations of fusion inhibitor T20 peptide [89] (AIDS Research and Reference Reagent Program) were incubated with TZM-bl cells and Env-variants for 48 hours, then, luciferase expression was measured (Bright-Glo, Promega) and the IC_50_ determined.

### Virus neutralization

Virus neutralization assays were performed using the following reagents: mAbs 4E10, 2 F5, and 2 G12 purchased from PolyMun; mAbs PG9 and PG16 provided by Dennis Burton; mAbs 19B and 17B provided by Barton Haynes and HIV-specific pool of IgG from HIV-1 clade C infected individuals with broad neutralization (HIVIG-C) obtained from David Montefiori. To examine sCD4 binding site neutralization sensitivity the following reagents were used sCD4 purchased from PolyMun, mAb IgG1B12 provided by Dennis Burton, CD4-Ig and mAbs VRC01, VRC03, VRC-PG04, NIH 45–46, 3BMC117, provided by John Mascola and mAb CH31 provided by Barton Haynes. Autologous virus neutralization was assessed with plasma and milk samples from CHAVI009 patients collected 4 to 6 weeks after delivery. The autologous neutralizing response against infant Env pseudoviruses was not assessed because maternal plasma and breast milk samples were not available. Prior to neutralization assessment, the plasma samples were heat inactivated at 56°C for 1 hour and the breast milk samples were delipidized by centrifugation at 25,000 g at 4°C for 30 minutes and filtered using spin X filter tubes (Fisher). The delipidized samples were then concentrated 4 fold using Amicon Ultra centrifugal filters with a 100 K cutoff. Neutralization was measured by reduction in luciferase reporter gene expression after a single round of infection in TZM-bl cells, as previously described [90]. The 50% inhibitory dose (ID_50_) titer was calculated as the plasma or milk dilution that caused a 50% reduction in relative luminescence units (RLU) compared to the virus control wells after subtraction of cell control RLUs. The 50% inhibitory concentration (IC_50_) titer was calculated as the reagent concentration that caused a 50% reduction in RLU.

### Statistical analysis

A mixed model was applied to compare the difference between the least square means of each virologic function of milk Env-variants of postnatal-transmitting women and nontransmitting women, and between postnatally-T/F infant Env variants and milk Env variants of nontransmitting women. This method takes into account multiple observations (virus variants) within each individual. In addition, Q values were calculated as a false discovery rate correction for multiple comparison [91]. Correlations between DC:virion interactions and glycosylation sites were performed with a Spearman’s rank correlation; and the Wilcoxon match-paired test was used to compare DC transfer efficiency between viruses produced in PBMC and 293 T cells. The Fisher’s exact test was used to compare the proportion of viruses that were sensitive to autologous neutralization.

## Competing interest

The authors declare that they have no competing interest.

## Authors’ contributions

GF conducted the neutralization assays, fusion inhibition and co-receptor usage assays, infectivity assays, virus production, epithelial binding assays and contributed to manuscript development. TM conducted the DC binding and transfer assays, Envelope cloning, virus production, Envelope proteins production and contributed to manuscript development. JS and MS contributed to cloning, sequencing and identification of infant T/F Env variants. GL developed the phylogenic trees. SK and JK did the DC-SIGN binding assay. MD and MA performed and analyzed the Galcer binding assay. ER and RS did the infant T/F Env cloning. KR contributed to the epithelial binding assay. FJ and MA did the Envelope protein purification. FC and FG contributed to the infant T/F Env cloning and sequencing. NV conducted the statistical analysis. BH and GS contributed to the breast milk Env cloning. CO produced the infant T/F IMCs. SM, VM and LK were clinical site investigators. SF oversaw testing infants for HIV infection. DM contributed to the analysis and interpretation of the neutralization data. BH contributed to the data interpretation. SP designed the study, oversaw clinical and laboratory aspects of the study, analyzed and interpreted the data, and contributed to the manuscript development. All authors read and approved the final manuscript.

## Supplementary Material

Additional file 1 Figure S1 HIV-1 env clones selected for functional characterization from genetically-diverse viral variants found in breast milk of eight chronically HIV-infected Malawian women (A-H). Maximum likelihood phylogenetic trees for full-length HIV *env* RNA sequences amplified by single genome amplification from milk (blue circles and stars) and plasma (red circles) samples taken within 4-6 weeks postpartum are depicted. Blue filled circles denote milk sequences from the left breast, while blue stars indicate milk sequences from the right breast. Env sequences from topologically distinct branches, and when possible, from clusters of identical or near identical sequences (inferred to define clonally-amplified variants) were cloned into an expression vector. Milk Env clones that were functional (arrows) in a standard pseudotyping assay were further characterized. Milk Env clones (2 to 5 per patient) from three transmitting (D, G, and H) and five nontransmitting (A, B, C, E, and F) women are depicted. In two transmitting women, the milk sequence that was identical (4403_BMC5) or closest (1209_BMH5) to the virus that infected the corresponding infant was identified and included in the study. Numbers at the nodes indicate the level of support for tree branches (likelihood ratio test values ≥0.095). The scale bar represents 0.01 substitutions per site. Click here for file

Additional file 2 Figure S2 No difference in DC-SIGN binding efficiency between T/F infant and breast milk Env virus variants. (A) DC-SIGN mediated trans-infection efficiency of milk and infant Env virus variants in the presence (black bar) and absence (grey bar) of mannan. While mannan treatment generally reduced viral transfer to indicator cells, a significant portion of the binding was not inhibited by mannan treatment. Bars represent the mean transfer efficiency of three replicates, error bars represent standard deviation. (B) Dot plot of the mannan-inhibitable DC-SIGN transfer efficiency of Env virus variants from transmitting and nontransmitting women, and postnatally-infected infants. The line represents median normalized DC-SIGN transfer efficiency for each Env-pseudovirus, grouped by transmission category. Stars represent transmitted milk variants. (PDF 326 kb) Click here for file
