# Supplementary material for: Postnatally-transmitted HIV-1 Envelope variants have similar neutralization-sensitivity and function to that of nontransmitted breast milk variants
Source: Retrovirology. 2013 Jan 10;10:3. doi: 10.1186/1742-4690-10-3 (PMC3564832; doi:10.1186/1742-4690-10-3)
Supplement: Additional file 1 Figure S1 — HIV-1 env clones selected for functional characterization from genetically-diverse viral variants found in breast milk of eight chronically HIV-infected Malawian women (A-H). Maximum likelihood phylogenetic trees for full-length HIV env RNA sequences amplified by single genome amplification from milk (blue circles and stars) and plasma (red circles) samples taken within 4-6 weeks postpartum are depicted. Blue filled circles denote milk sequences from the left breast, while blue stars indicate milk sequences from the right breast. Env sequences from topologically distinct branches, and when possible, from clusters of identical or near identical sequences (inferred to define clonally-amplified variants) were cloned into an expression vector. Milk Env clones that were functional (arrows) in a standard pseudotyping assay were further characterized. Milk Env clones (2 to 5 per patient) from three transmitting (D, G, and H) and five nontransmitting (A, B, C, E, and F) women are depicted. In two transmitting women, the milk sequence that was identical (4403_BMC5) or closest (1209_BMH5) to the virus that infected the corresponding infant was identified and included in the study. Numbers at the nodes indicate the level of support for tree branches (likelihood ratio test values ≥0.095). The scale bar represents 0.01 substitutions per site. [file 1742-4690-10-3-S1.pdf]

Figure S1.

HIV-1 env clones selected for functional characterization from genetically-diverse viral variants found in breast milk of eight chronically HIV-infected Malawian women (A-H). Maximum likelihood phylogenetic trees for full-length HIV *env* RNA sequences amplified by single genome amplification from milk (blue circles and stars) and plasma (red circles) samples taken within 4-6 weeks postpartum are depicted. Blue filled circles denote milk sequences from the left breast, while blue stars indicate milk sequences from the right breast. Env sequences from topologically distinct branches, and when possible, from clusters of identical or near identical sequences (inferred to define clonally-amplified variants) were cloned into an expression vector. Milk Env clones that were functional (arrows) in a standard pseudotyping assay were further characterized. Milk Env clones (2 to 5 per patient) from three transmitting (D, G, and H) and five nontransmitting (A, B, C, E, and F) women are depicted. In two transmitting women, the milk sequence that was identical (4403_BMC5) or closest (1209_BMH5) to the virus that infected the corresponding infant was identified and included in the study. Numbers at the nodes indicate the level of support for tree branches (likelihood ratio test values ≥0.095). The scale bar represents 0.01 substitutions per site.

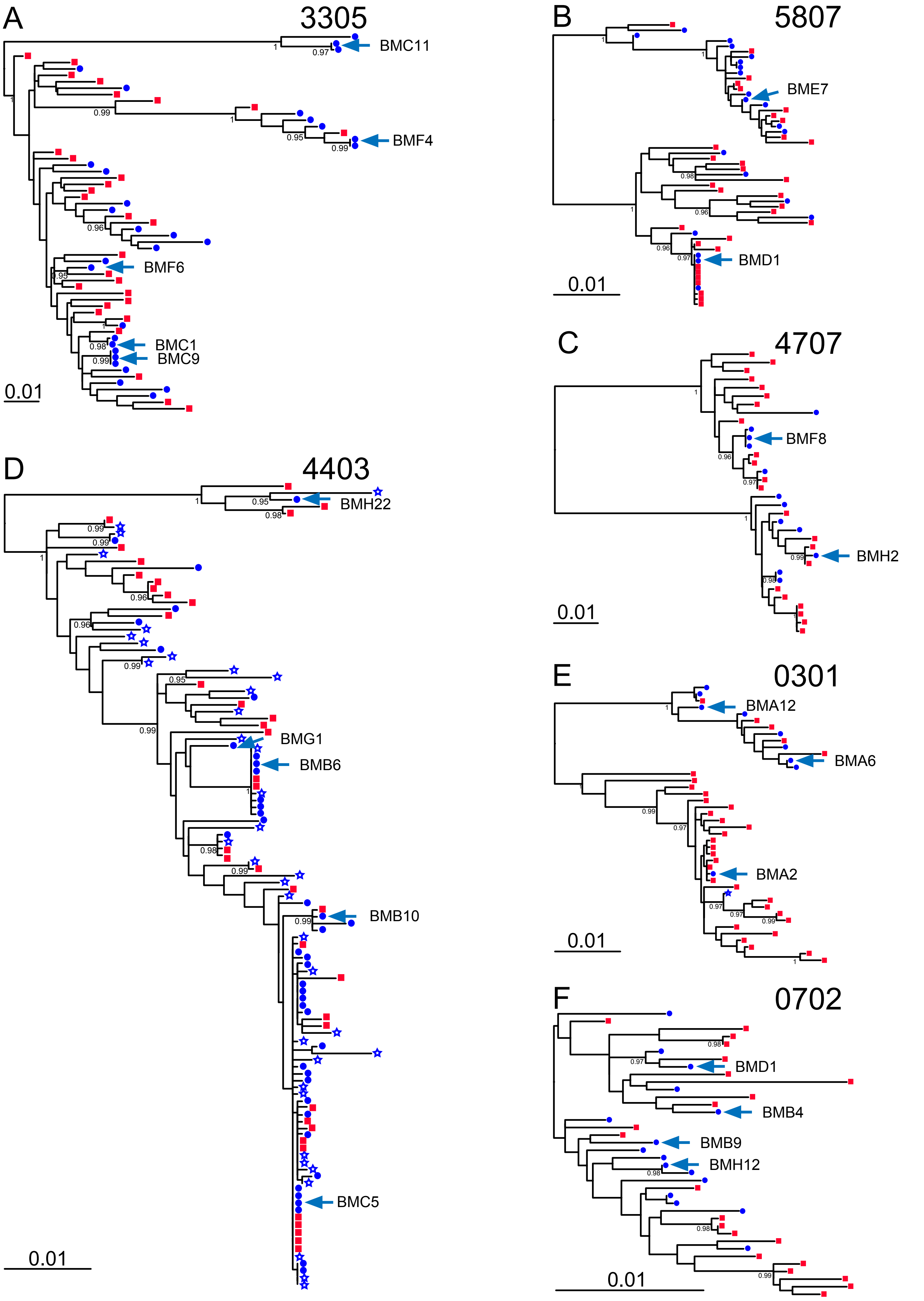
A
3305
BMC11
BMF4
BMF6
BMC1
BMC9
0.01
B
5807
BME7
BMD1
0.01
C
4707
BMF8
BMH2
0.01
D
4403
BMH22
BMG1
BMB6
BMB10
BMC5
0.01
E
0301
BMA12
BMA6
BMA2
0.01
F
0702
BMD1
BMB4
BMB9
BMH12
0.01

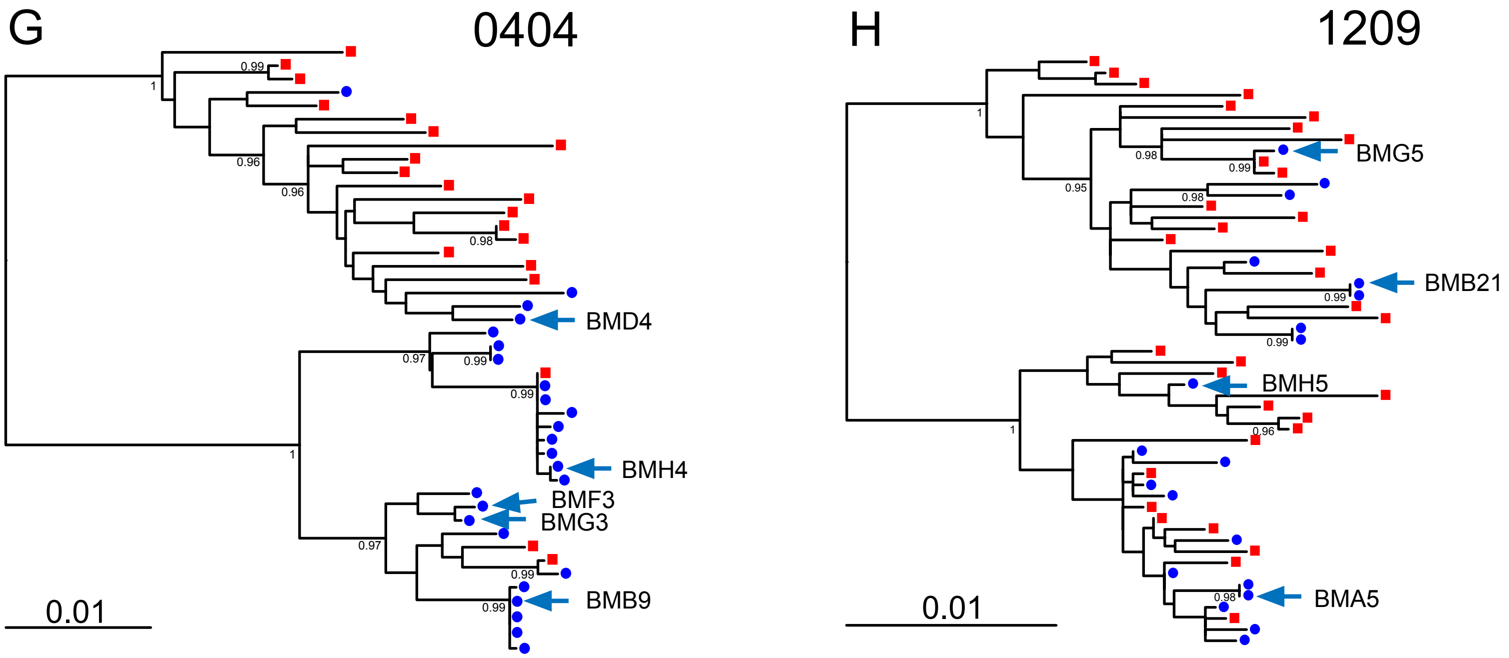

G
0404
BMD4
BMH4
BMF3
BMG3
BMB9
0.01
H
1209
BMG5
BMB21
BMH5
BMA5
0.01
