# Supplementary material for: Postnatally-transmitted HIV-1 Envelope variants have similar neutralization-sensitivity and function to that of nontransmitted breast milk variants
Source: Retrovirology. 2013 Jan 10;10:3. doi: 10.1186/1742-4690-10-3 (PMC3564832; doi:10.1186/1742-4690-10-3)
Supplement: Additional file 2 Figure S2 — No difference in DC-SIGN binding efficiency between T/F infant and breast milk Env virus variants. (A) DC-SIGN mediated trans-infection efficiency of milk and infant Env virus variants in the presence (black bar) and absence (grey bar) of mannan. While mannan treatment generally reduced viral transfer to indicator cells, a significant portion of the binding was not inhibited by mannan treatment. Bars represent the mean transfer efficiency of three replicates, error bars represent standard deviation. (B) Dot plot of the mannan-inhibitable DC-SIGN transfer efficiency of Env virus variants from transmitting and nontransmitting women, and postnatally-infected infants. The line represents median normalized DC-SIGN transfer efficiency for each Env-pseudovirus, grouped by transmission category. Stars represent transmitted milk variants. (PDF 326 kb) [file 1742-4690-10-3-S2.pdf]

S2: No difference in DC-SIGN binding efficiency between T/F infant and breast milk Env virus variants. (A) DC-SIGN mediated trans-infection efficiency of milk and infant Env virus variants in the presence (black bar) and absence (grey bar) of mannan. While mannan treatment generally reduced viral transfer to indicator cells, a significant portion of the binding was not inhibited by mannan treatment. Bars represent the mean transfer efficiency of three replicates, error bars represent standard deviation. (B) Dot plot of the mannan-inhibitable DC-SIGN transfer efficiency of Env virus variants from transmitting and nontransmitting women, and postnatally-infected infants. The line represents median normalized DC-SIGN transfer efficiency for each Env-psuedovirus, grouped by transmission category. Stars represent transmitted milk variants.

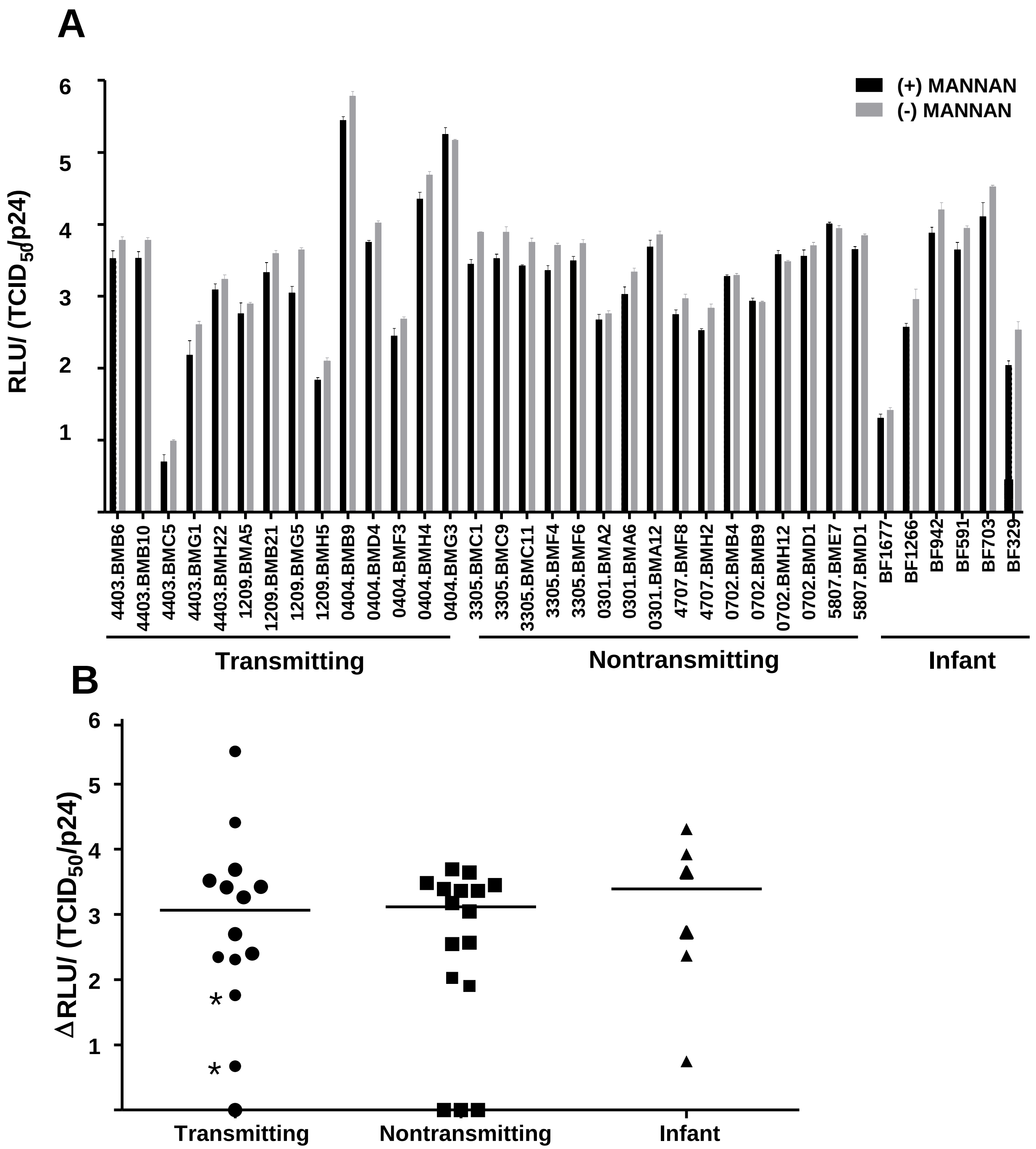
A
(+) MANNAN
(-) MANNAN
RLU/ (TCID50/p24)
4403.BMB6
4403.BMB10
4403.BMC5
4403.BMG1
4403.BMH22
1209.BMA5
1209.BMB21
1209.BMG5
1209.BMH5
0404.BMB9
0404.BMD4
0404.BMF3
0404.BMH4
0404.BMG3
3305.BMC1
3305.BMC9
3305.BMC11
3305.BMF4
3305.BMF6
0301.BMA2
0301.BMA6
0301.BMA12
4707.BMF8
4707.BMH2
0702.BMB4
0702.BMB9
0702.BMH12
0702.BMD1
5807.BME7
5807.BMD1
BF1677
BF1266
BF942
BF591
BF703
BF329
Transmitting
Nontransmitting
Infant
B
ΔRLU/ (TCID50/p24)
Transmitting
Nontransmitting
Infant
